# Sedentary behavior from television watching elevates GlycA levels: A bidirectional Mendelian randomization study

**DOI:** 10.1371/journal.pone.0308301

**Published:** 2024-08-01

**Authors:** Shuchuan Miao, Xiaoyan Wang, Lu Ma, Chao You

**Affiliations:** 1 Department of Neurosurgery, West China Hospital, Sichuan University, Chengdu, China; 2 Department of Neurosurgery, Chengdu Seventh People’s Hospital, Chengdu, China; 3 Department of Clinical Nutrition, The First Affiliated Hospital of Chengdu Medical College, Chengdu, China; University of Costa Rica, COSTA RICA

## Abstract

**Background:**

Current evidence linking sedentary behavior (SB), physical activity (PA), and inflammation raises questions about their causal relationships, prompting concerns about potential residual confounding or reverse causation.

**Methods:**

A bidirectional Mendelian randomization (MR) analysis was conducted. SB data (n = 408,815) from “computer use,” “television watching,” and “driving” were included. The PA data encompassed nine types of PA (n = 460,376) over the last four weeks and included data on the frequency of vigorous PA (n = 440,512) and moderate PA (n = 440,266) for over 10 min. Additionally, three genome-wide association study datasets (n = 64,949) on light, moderate, and vigorous exercise were included to minimize potential bias from changes in exercise intensity. Inflammation data included levels of C-reactive protein (CRP) (n = 575,531), glycoprotein acetyl (GlycA) (n = 115,082), interleukin (IL)-8, IL-6, IL-6 receptor (IL-6R), and soluble IL-6R (sIL-6R) (n = 35,278). All datasets represented participants of European ancestry.

**Results:**

Television watching as an SB showed significant positive causal effects on GlycA and CRP (inverse variance weighted (IVW), odds ratios (OR): 1.34, 95% confidence intervals (CI): 1.25–1.44, *p* = 3.570 × 10^−17^; IVW, OR: 1.21, 95% CI: 1.16–1.26, *p* = 1.500 × 10^−19^, respectively), with more robust evidence for GlycA. In the direction from inflammation to PA, a negative causal relationship between CRP and“number of days/week of moderate PA 10+ minutes”was observed (IVW, OR: 0.92, 95% CI: 0.89–0.96, *p* = 3.260 × 10^−5^). Sensitivity analyses were used to verify the robustness and reliability of the results. However, other initially observed associations ceased to be significant after controlling for obesity-related confounders.

**Conclusion:**

Our MR analysis suggested a potential causal relationship between television watching and chronic low-grade inflammation, with more substantial evidence for GlycA. Additionally, different types of SB may have varying effects on inflammation. Obesity-related traits could partly or entirely influence the relationship between SB, PA, and inflammatory markers. Furthermore, Our findings indicate that SB is an independent risk factor for inflammation, separate from PA, and highlight the different mechanisms by which SB and PA affect disease.

## Introduction

Inflammation is the response of the host to defend against toxins, bacteria, viruses, tissue damage, and metabolic stress, mobilizing immune and non-immune cells [1, 2]. A typical inflammatory response involves a transient increase in inflammatory activity in the presence of a threat, which diminishes upon resolution [2–4]. However, prolonged inflammation can lead to chronic pathological conditions when the relevant regular mechanisms cease to exert their influence [5]. Chronic low-grade inflammation precedes various chronic disorders, such as type 2 diabetes, obesity, heart disease, and cancer. It is marked by a 2–4 fold increase in circulating proinflammatory factors (such as interleukins (IL)-6, IL-8, tumor necrosis factor-α), acute-phase proteins (like C-responsive protein (CRP) and fibrinogen-activating factor), and anti-inflammatory cytokines (such as interleukins-10 (IL-10)). This state of chronic inflammation has been linked to various lifestyle factors, and emerging evidence suggests that these inflammatory markers may be associated with increased sedentary behavior (SB) or reduced physical activity (PA) [6–8].

SB refers to activities involving energy expenditure ≤ 1.5 metabolic equivalent units while awake, typically in a seated or lying position, as defined [9]. Conversely, PA encompasses bodily movements that engage skeletal muscles, causing energy expenditure above resting levels [6]. Typical SB activities include computer use, television watching, and driving, which often involve prolonged sitting [10]. SB is a significant aspect of human activity that can negatively affect overall health [11, 12]. Recent research indicates an association between SB or PA and inflammatory markers, such as CRP [13–15], IL-6 [13–17], and IL-8 [18], although the results of specific studies present inconsistencies [18–21]. The current evidence connecting SB, PA, and inflammation raises questions about their causal relationships, which are potentially influenced by residual confounding or reverse causation. Addressing these questions through randomized controlled trials (RCTs) is often challenging because RCTs can be costly, time-consuming, and sometimes ethically or practically challenging, particularly when studying long-term exposures or rare outcomes [22]. However, observational cohort and cross-sectional studies are available as alternatives, but they cannot definitively establish causality and are susceptible to confounding [23].

Mendelian randomization (MR) offers an alternative method to explore cause-and-effect relationships using genetic variations as a natural experiment. This approach categorizes individuals into groups with differing mean levels of nongenetic exposure over their lifetimes. Genetic variants are assigned randomly before birth, independent of environmental factors or lifestyle choices, which typically confound observational studies [24]. This randomness allows researchers to study the effects of genetic variations on health outcomes as if the subjects were randomly assigned different levels of exposure to a particular risk factor [25]. Thus, MR provides a way to overcome issues such as confounding variables and reverse causation, which are common challenges in traditional observational research [26].

This study utilized a two-sample MR analysis to investigate the potential causal relationship between SB, PA, and inflammatory markers (CRP, IL-6, IL-6 receptor (IL-6R), soluble IL-6R (sIL-6R), and IL-8). We included the newly introduced inflammatory marker, glycoprotein acetylation (GlycA), known for its enhanced stability and robust association with chronic low-grade inflammation [27–29]. We conducted bidirectional analyses to examine these possibilities.

## Methods

### Study design

**Fig 1:**
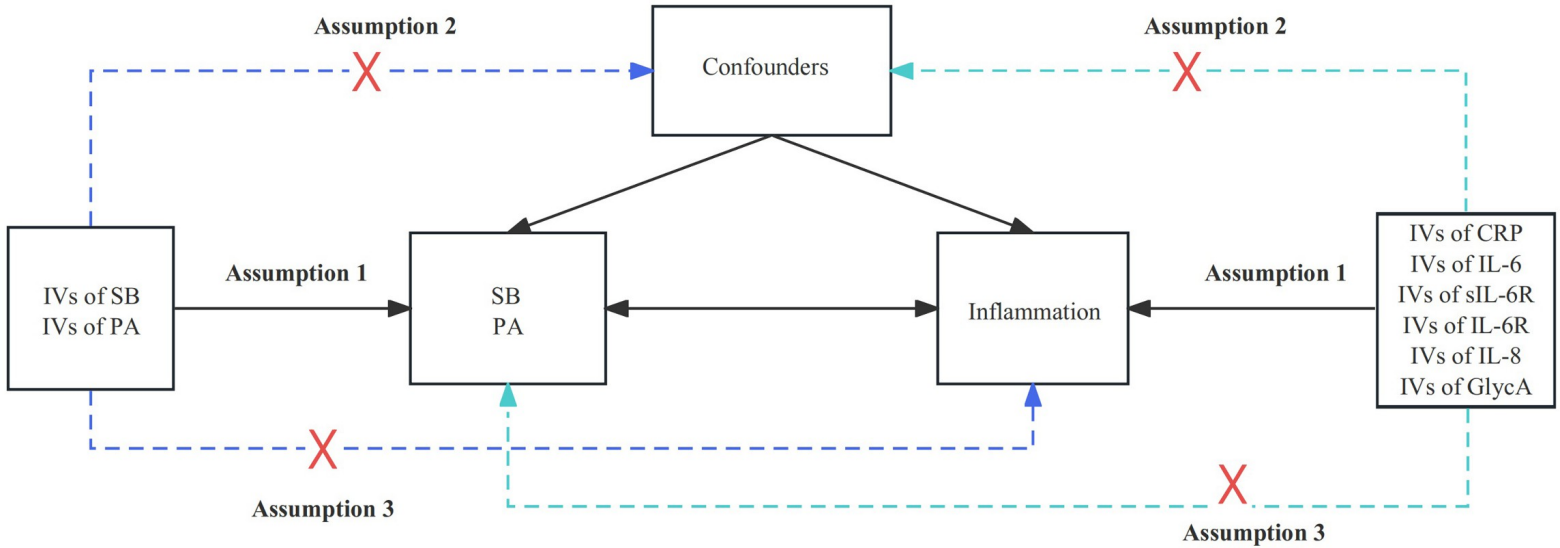
Flowchart of the bidirectional MR study between SB, PA, and inflammation. MR analysis relies upon three key assumptions: 1. The IVs should exhibit a significant association with the exposure under investigation. 2. The IVs should not be associated with any risk factors related to the exposure-outcome relationship. 3. The IVs should exclusively influence the outcome through their impact on the exposure. IVs: instrumental variables, SB: sedentary behaviour, PA: physical activity, CRP: C-reactive protein, GlycA: glycoprotein acetyls, IL-6: interleukin-6, IL-6R: IL-6 receptor, sIL-6R: soluble IL-6R, IL-8: interleukin-8.

This bidirectional MR study employed extensive genome-wide association studies (GWAS) summary datasets as a secondary analysis of publicly available data. Ethical approval for the original GWAS is outlined in their respective publications. The MR analysis is based on three key assumptions. (1) The instrumental variables (IVs) must be strongly associated with exposure to interest. (2) IVs should not be connected to any risk factors related to the exposure–outcome relationship. (3) IVs should influence outcomes solely through their effect on the exposure [30, 31]. We initially selected the genetic IVs associated with SB and PA to infer the causal relationships between SB, PA, and inflammation (Fig 1). Subsequently, we used genetic IVs associated with inflammation to establish causality between inflammation and SB and PA (Fig 1).

### Data sources

The GWAS listed in Table 1 were used to identify genetic variants associated with SB, PA, and inflammatory markers (GlycA, CRP, IL-6, IL-6R, sIL-6R, and IL-8). Van de Vegte YJ et al. recently conducted three GWAS on SB, examining time spent in “computer use,” “television watching,” and “driving,” involving 408,815 participants [32]. The duration of each of the three behaviors was assessed by the participant’s responses to the following question: “On a typical day, how many hours do you spend watching television?” “On a typical day, how many hours do you spend using a computer? (Do not include using a computer at work),” and “On a typical day, how many hours do you spend driving?” Mean reported times from the original study were 2.8 h (standard deviation [SD] 1.5 h) for television watching, 1.0 h (SD 1.2 h) for computer use, and 0.9 h (SD 1.0 h) for driving. Genetic variants associated with SB were controlled for multiple covariates, including age, sex, body mass index (BMI), smoking status, hypertension, diabetes, Townsend deprivation index, PA levels, weekly alcohol consumption, and educational attainment [32].

**Table 1:** Detail of the data for the cohort population.

Variable	Samplesize(cases/controls)	Data resource	PMID	Year
**SB**				
Television watching	408,815(NA/NA)	GCST010084	32317632	2020
Computer use	408,815(NA/NA)	GCST010085	32317632	2020
Driving	408,815(NA/NA)	GCST010086	32317632	2020
**PA**				
**Types of PA in last 4 weeks**				
Light DIY	460,376(236,244/224,132)	ukb-b-11495	NA	2018
Heavy DIY	460,376(197,006/263,370)	ukb-b-13184	NA	2018
Walking for pleasure	460,376(329,755/130,621)	ukb-b-7337	NA	2018
Strenuous sports	460,376(47,468/412,908)	ukb-b-7663	NA	2018
Other exercises	460,376(222,470/237,906)	ukb-b-8764	NA	2018
None of the above	460,376(28,040/432,336)	ukb-b-15869	NA	2018
**Number of days/week of**				
Vigorous PA 10+ minutes	440,5 12(NA/NA)	ukb-b-151	NA	2018
Moderate PA 10+ minutes	440,266(NA/NA)	ukb-b-4710	NA	2018
**Time spent doing**				
Light PA	64,949(NA/NA)	ukb-b-8865	NA	2018
Moderate PA	64,949(NA/NA)	ukb-b-2115	NA	2018
Vigorous PA	64,949(NA/NA)	ukb-b-13702	NA	2018
**Inflammation**				
CRP	575,531(NA/NA)	GCST90029070	35459240	2022
GlycA	115,082(NA/NA)	GCST90092821	35213538	2022
IL-6	35,278(NA/NA)	NA	34857953	2021
IL-6 R	35,278(NA/NA)	NA	34857953	2021
sIL-6 R	35,278(NA/NA)	NA	34857953	2021
IL-8	35,278(NA/NA)	NA	34857953	2021

SB: sedentary behaviour, PA: physical activity, CRP: C-reactive protein, GlycA: glycoprotein acetyls, IL-6: interleukin-6, IL-6R: IL-6 receptor, sIL-6R: soluble IL-6R, IL-8: interleukin-8, DIY: do-it-yourself, IVW: inverse variance weighted, NA: not available.

We sourced PA data from the IEU Open GWAS. The types of PA encompassed light “do-it-yourself” (DIY, such as pruning and watering the lawn, 236,244 cases/224,132 controls), heavy DIY (such as weeding, lawn mowing, carpentry, and digging 197,006 cases/263,370 controls), walking for pleasure (not as a means of transport, 329,755 cases/130,621 controls), strenuous sports (sports that make you sweat or breathe hard, 47,468 cases/412,908 controls), other exercises (such as swimming, cycling, keeping fit, and bowling, 222,470 cases/237,906 controls), none of the above (physical inactivity, 28,040 cases/432,336 controls) over the past four weeks. The GWAS data “number of days/week of vigorous PA 10+ minutes” involved 440,512 European samples from the UK Biobank, while data on “number of days/week of moderate PA 10+ minutes” was sourced from 440,266 UK Biobank participants. To address exercise intensity variations and ensure robustness, our analysis included three GWAS datasets covering light, moderate, and vigorous exercise durations, totaling 64,949 participants. CRP data were sourced from Said et al. [33] (575,531 participants) and GlycA data from Richardson et al. [34] (115,082 participants). IL-8, IL-6, IL-6R, and sIL-6R data were obtained from Ferkingstad et al. [35] (35,278 participants), all involving individuals of European ancestry.

### Selection of genetic IVs

In adherence to MR assumptions (Fig 1), we identified single-nucleotide polymorphisms (SNPs) from published GWAS datasets with genome-wide significance (*p* < 5 × 10^−8^) [36] that robustly and independently predicted exposures while also demonstrating low linkage disequilibrium (R^2^ < 0.001 within 10 Mb) [37]. A fundamental MR assumption necessitates that SNPs affect the outcome exclusively through the exposure under investigation. To confirm this, we evaluated their relationships with potential confounders, specifically obesity-related traits such as BMI, waist circumference, hip circumference, waist-to-hip ratio, and body fat-related traits, using PhenoScanner (www.phenoscanner.medschl.cam.ac.uk, accessed on November 11, 2023), a platform offering extensive genotype-phenotype association data [38]. SNPs associated with potential confounders of genome-wide significance were controlled [39]. Finally, we evaluated SNP strength by considering the mean F-statistic, where a mean F-statistic > 10 suggests adequate power, supporting the validity of the SNPs for the trait [40].

### Statistical analyses

We conducted a bidirectional two-sample MR analysis utilizing summary data to explore the causal relationships between SB, PA, and inflammation. Our methodology involves several pivotal steps.

1. Genetic IV selection: We performed SNP clumping to derive independent genetic instruments, eliminating SNPs significantly associated with the outcomes. Ambiguous and palindromic SNPs were also excluded.
2. MR analysis: MR analysis utilized the IVW method, presenting results as odds ratios (OR) and 95% confidence intervals (95% CI) [41]. Four other methods were also used: MR-Egger, weighted median, weighted mode, and MR-PRESSO.
3. Directionality confirmation: To verify the causality direction in univariate MR, we utilized the MR-Steiger directionality test and performed a reverse MR analysis.
4. Sensitivity analysis: This included heterogeneity testing and pleiotropy assessment. We used Cochran’s Q test to detect heterogeneity in the IVW approach and the MR-Egger intercept to identify horizontal pleiotropy (an intercept with *p* < 0.05 indicating the presence of horizontal pleiotropy) [42, 43]. Cochran’s Q test indicated significant heterogeneity (*p* < 0.05), prompting the use of a random effects model for the remaining studies. Additionally, we conducted a leave-one-out analysis to explore the influence of each genetic variant on the outcome [43] and applied MR-PRESSO to detect and correct potential pleiotropic biases [44]. Our interpretation criteria considered IVW estimates to have causal associations if they were consistent in direction and significance with at least one sensitivity analysis and showed no evidence of pleiotropy (*p* > 0.05). We reported effect sizes as OR, beta values, proportions, and their corresponding 95% CI. Among the positive results, we excluded IVs associated with the outcome and potential confounders, such as BMI, waist circumference, hip circumference, waist-hip ratio, and body fat-related characteristics (S1 Table in S1 File), using PhenoScanner. In this study, we analyzed 6 inflammatory markers, 3 subtypes of SB, and 11 subtypes of PA. We applied Bonferroni correction to avoid false-positive results in two-way multiple tests, considering a significance level of *p* < 0.05/number of exposures/number of outcomes. All analyses were conducted using the TwoSampleMR (version 0.5.7) and MRPRESSO packages (version 1.0.0) in R Software (version 4.3.1) (https://www.R-project.org).

## Results

### Causal effects of SB and PA on inflammation

#### Television watching and inflammation

**Fig 2:**
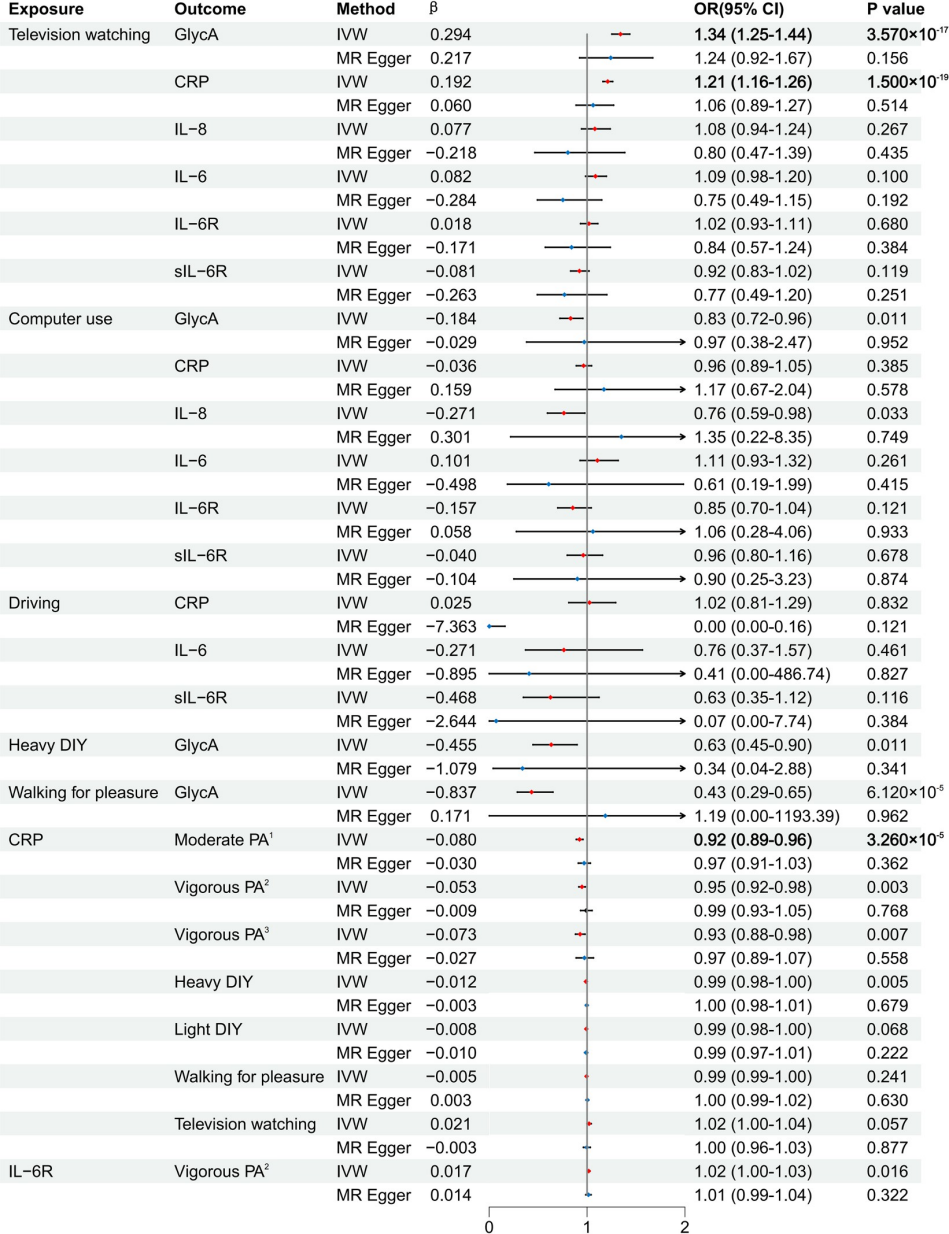
Main results on the causal relationships between SB, PA, and inflammation. GlycA: glycoprotein acetylation, CRP: C-responsive protein, PA: physical activity, OR: odds ratios, CI: confidence intervals, IL-6: interleukin-6, IL-6R: IL-6 receptor, sIL-6R: soluble IL-6R, IL-8: interleukin-8, MR: mendelian randomization, DIY: do-it-yourself, IVW: inverse variance weighted.

**Fig 3:**
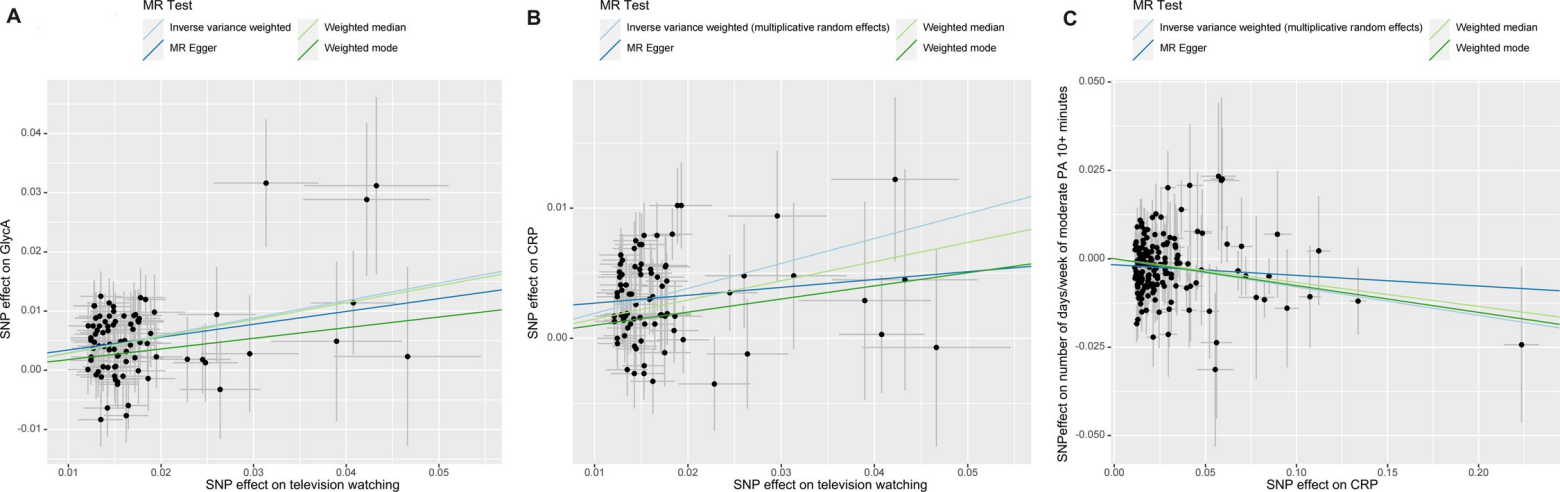
Scatter plots of television watching to GlycA, CRP, and CRP to “number of days/week of moderate PA 10+ minutes”. (A) Scatter plot of television watching to GlycA. (B) Scatter plot of television watching to CRP. (C) Scatter plot of CRP to “number of days/week of moderate PA 10+ minutes.” GlycA: glycoprotein acetylation, CRP: C-responsive protein, PA: physical activity, SNP: single nucleotide polymorphism, MR: mendelian randomization.

Television watching showed significant positive causal effects on GlycA and CRP levels. Specifically, a 1.5-h increase in television watching was associated with a 34% increase in GlycA (IVW, OR: 1.34, 95% CI: 1.25 to 1.44, *p* = 3.57 × 10^−16^) and a 21% increase in CRP (IVW, OR: 1.21, 95% CI: 1.16–1.26, *p* = 1.50 × 10^−18^) (Fig 2). These results were consistent in the beta direction across different MR methods, including MR-Egger, weighted median, and weighted mode approaches, and were significant in the weighted median approaches, confirming the robustness of our findings (Figs 2 and 3A and 3B). However, we did not observe a correlation between television watching and IL-8, which ceased to be significant when controlled for obesity-related confounders (IVW, OR: 1.08, 95% CI: 0.94–1.24, *p* = 0.27) (Fig 2). Furthermore, there was no observed correlation between television watching and other inflammatory markers such as IL-6 (IVW, OR: 1.09, 95% CI: 0.98–1.20, *p* = 0.10), IL-6R (IVW, OR: 1.02, 95% CI: 0.93–1.11, *p* = 0.68), and sIL-6R (IVW, OR: 0.92, 95% CI: 0.83–1.02, *p* = 0.12) (Fig 2 and S2 Table in S1 File). This indicates that the impact of television watching may be specific to certain inflammatory markers such as CRP and GlycA.

#### Computer use, driving, and inflammation

Correlations between computer use and IL-8, IL-6R, and GlycA levels showed inconsistent direction or significance after controlling for obesity-related confounders (Fig 2). No causal relationships were found between computer use or driving and the inflammatory markers CRP, IL-6, or sIL-6R (Fig 2). These findings indicate that different types of SB do not have the same effect on inflammation, highlighting the distinct impact of television watching.

#### PA and inflammation

While none of the estimates survived Bonferroni correction (S3 Table in S1 File), caution is advised regarding estimates with P_IVW_ < 0.05 and consistent beta direction. There was a potential causal correlation between heavy DIY and GlycA (IVW, OR: 0.63, 95% CI: 0.45–0.90, *p* = 0.01 > 0.05/11/6) (Fig 2). Similarly, walking for pleasure showed a potential causal correlation with reduced GlycA levels, although these associations were inconsistent after controlling for obesity-related confounders (Fig 2). These findings indicate that certain types of PA may help to reduce specific inflammatory markers.

### Causal effects of inflammation on SB and PA

CRP levels showed a significant negative causal relationship with “number of days/week of moderate PA 10+ minutes” (IVW, OR: 0.92, 95% CI: 0.89–0.96, *p* = 3.26 × 10^−5^) (Fig 2). This relationship was consistent in the beta direction across various sensitivity analyses and was significant in the weighted median and weighted mode approaches (Figs 2 and 3C), indicating that higher CRP levels were associated with reduced levels of moderate PA.

Further analyses revealed that increased CRP levels were also associated with light DIY activities, walking for pleasure, and television watching, although these associations became less robust after controlling for obesity-related confounders (Fig 2 and S4 and S5 Tables in S1 File). While no estimates survived Bonferroni correction (0.05/6/14, S4 Table in S1 File), estimates with P_IVW_ < 0.05 and consistent beta direction suggest potential causal correlations between CRP and “number of days/week of vigorous PA 10+ minutes,” heavy DIY activities, time spent engaging in vigorous PA, as well as between IL-6R and “number of days/week of vigorous PA 10+ minutes”(Fig 2).

### Sensitivity analyses

To ensure the validity of the genetic IVs, we conducted various sensitivity analyses. Initially, 147 genetic variants were identified for the impact of television watching on GlycA, but 57 SNPs were controlled due to potential confounding by obesity-related traits, such as BMI, waist circumference, hip circumference, waist-hip ratio, and body fat-related characteristics. MR-PRESSO identified 1 outlier SNP, and the Steiger test filtered out 3 invalid variants (S6 Table in S1 File). After removing these 61 SNPs, the MR estimates were consistent with the previous findings, indicating that, while confounders attenuated the causal relationship between television watching and GlycA, they did not violate (Fig 2 and S2 Table in S1 File). The F-statistics ranged from 28.8 to 74.8, with a mean of 38.43, indicating a robust predictive instrument capacity for television watching (S7 Table in S1 File). No significant heterogeneity or horizontal pleiotropy was detected (IVW, Q value = 98.9, Q df = 85, Q *p*-value = 0.14; MR’s Q = 98.5, Q df = 84, Q *p*-value = 0.13; Table 2). For the effects of television watching on CRP, 126 genetic variants were initially identified, with 43 SNPs controlled for obesity-related traits and 1 outlier identified by MR-PRESSO, resulting in a mean F-statistic of 37.88 (S8 and S9 Tables in S1 File). Significant heterogeneity was observed; however, no horizontal pleiotropy was identified (Table 2). Analyzing CRP in relation to “number of days/week of moderate PA 10+ minutes,” the F-statistics ranged from 23.3 to 1979.9 (S10 Table in S1 File). Moreover, subsequent analyses showed heterogeneity, yet no evidence of horizontal pleiotropy was observed (Table 2). The leave-one-out analysis showed that no single SNP affected the results. S1–S3 Figs illustrate the forest plots, leave-one-out plots, and funnel plots, respectively.

**Table 2:** Heterogeneity and pleiotropy test results for television watching, GlycA, CRP, and moderate PA.

Direction	Test	Q value	Q df	Q p-value	MR Egger Intercept	SE	P value
Television watching to GlycA	IVW^2^	98.9	85	0.14			
	MR Egger^2^	98.5	84	0.13			
	MR Egger^3^				0.001	0.002	0.6
Television watching to CRP	IVW^2^	133.04	81	0.0002			
	MR Egger^2^	129.6	80	0.0004			
	MR Egger^3^				0.002	0.001	0.14
CRP to moderate PA^1^	IVW^2^	192.98	159	0.03			
	MR Egger^2^	188.82	158	0.05			
	MR Egger^3^				-0.002	0.0009	0.06

^1^ Number of days/week of moderate PA 10+ minutes

^2^Heterogeneity

^3^Horizontal pleiotropy

PA: physical activity, CRP: C-reactive protein, GlycA: glycoprotein acetyls, IVW: inverse variance weighted, MR:

mendelian randomization.

## Discussion

Using a two-sample bidirectional MR approach, we rigorously examined the bidirectional causal relationship between different types of SB, PA, and inflammation. Regarding the direction of SB on inflammation, our study identified a causal effect of television watching on GlycA and CRP levels. This indicates a potential causal relationship between television watching and chronic low-grade inflammation, with more substantial indications for GlycA. Furthermore, examining the influence of inflammation on PA, our study revealed the causal impact of CRP on “number of days/week of moderate PA 10+ minutes.” Although associations between CRP levels and light DIY activities, walking for pleasure, and television watching were initially observed, they became nonsignificant after controlling for potential confounders. Additionally, our MR analysis showed that computer use, heavy DIY activities, and walking for pleasure were associated with GlycA; television watching was associated with IL-8; and computer use was associated with IL-8, IL-6R, and GlycA. However, these associations were entirely explained by the potential confounders associated with obesity-related traits.

Recent clinical studies have shown that SB and PA are associated with GlycA levels. GlycA, a novel spectroscopic marker of systemic inflammation, demonstrates low intraindividual variability and is favorable for potential clinical applications in patients with chronic inflammatory and autoimmune conditions [29]. These findings indicate that GlycA may more comprehensively reflect systemic inflammation than CRP, a commonly used traditional inflammatory marker [45]. A nested case-control study within the prospective China Kadoorie Biobank, involving 3,195 cases of cardiovascular disease onset and 1,465 control cases, revealed that after controlling for confounders, SB exhibited a positive correlation with the inflammatory marker GlycA, whereas PA displayed an inverse correlation with GlycA [46]. The study indicates that GlycA alone might account for 25% of the total effect of PA on cardiovascular disease (CVD), implying that inflammatory pathways may serve as a promising mechanism for elucidating the protective association between PA and CVD. Additionally, a prospective cohort study involving 1,826 male and female participants revealed a negative correlation between moderate-to-vigorous PA and changes in sedentary time and GlycA [47]. A study involving 390 children investigated the association between PA, SB, diet quality, and inflammatory biomarkers. Initially, there was an inverse association between PA and GlycA after controlling for age and sex. However, these associations ceased to be statistically significant after further controlling for body fat percentage [48]. Contrary to clinical findings, our MR analysis revealed a positive causal association between SB from television watching and GlycA. However, no correlation was found between driving as an SB and GlycA.

Moreover, after controlling for confounders, the negative associations between SB from computer use, PA, and GlycA levels became less pronounced. Television watching, characterized by low muscle activity and energy expenditure, contrasts with computer use, which typically involves more vital cognitive stimulation. This cognitive engagement during computer use may mitigate the adverse physiological effects of SB [9, 49, 50]. Hence, accurately categorizing SB is crucial for understanding its potential causal or correlational relationships with GlycA.

Current research suggests that SB and PA are associated with CRP, IL-6, IL-8, and IL-10 levels, although the results of some studies are inconsistent. Some studies have demonstrated that prolonged periods of SB increase specific inflammatory markers, such as plasma concentrations of CRP and IL-6, in adults and older participants [14–17]. Conversely, after controlling for other inflammatory markers, PA displayed an inverse association with plasma concentrations of IL-6 and CRP [13]. Moreover, a longitudinal study involving healthy older adults found that a 14-d reduction in daily steps (70–76% reduction from baseline) led to elevated plasma concentrations of CRP [8, 9] and IL-6 [5]. Furthermore, in children and adolescents, SB showed a direct correlation, whereas PA displayed an inverse relationship with high-sensitivity CRP (hs-CRP) and IL-6 [48, 51–54]. However, three other studies reported no change in IL-6 levels after 7–8 h of uninterrupted sitting in healthy adults [20], adults with overweight or obesity [55], and postmenopausal females with rheumatoid arthritis [19].

Furthermore, another study observed substantial reductions (approximately 91%) in hs-CRP concentrations after 6 h of prolonged, uninterrupted sitting [20]. Additionally, research by Dorneles et al. revealed that high-intensity interval exercise decreases systemic IL-8 levels while increasing IL-6 and IL-10 levels [18]. Evidence suggests associations between SB, PA, and inflammatory markers (CRP, IL-6, IL-8, and IL-10); however, causality and direction remain uncertain. Previous studies predominantly concentrated on common indicators, such as IL-6 and CRP, which, despite their utility in assessing systemic inflammation, demonstrate temporal variability at individual levels [56]. Our MR analysis showed a positive causal relationship between television watching and CRP. Additionally, our findings indicated a negative causal relationship between CRP and the “number of days/week of moderate PA 10+ minutes,” though the mechanism behind this remains unclear and may involve unspecified factors. However, we found no correlations between SB, PA, and IL-10 levels. Additionally, we found no causal correlation between SB and IL-6, IL-6R, or sIL-6R levels. This could be attributed to the possibility that increased IL-6R expression might partly counteract IL-6 downregulation [57].

Our study has several implications. First, it demonstrates a positive causal relationship between television watching and the inflammatory markers GlycA and CRP. Conversely, there is a negative correlation between computer use and inflammation, indicating variability in SB types and their effects on the body, which may even be reversed. Second, after controlling for confounders, the associations between computer use, PA, and inflammation became nonsignificant, indicating that obesity-related traits may predominantly drive these correlations. Third, our findings delineate SB as an independent risk factor for inflammation, distinct from PA, offering insights into the various mechanisms underlying the effects of SB and PA on the body. Fourth, GlycA has emerged as a more robust biomarker for chronic low-grade inflammation than CRP, warranting further exploration as a potential biomarker for SB.

MR studies are less costly and more feasible than RCTs, particularly for investigating long-term health outcomes and exposures that are difficult to manipulate experimentally. Assigning participants to harmful levels of inactivity in an RCT to study SB, such as prolonged television watching, computer use, or driving, would be unethical. MR overcomes these ethical constraints by using genetic variants for these behaviors, allowing for investigation without directly exposing the participants to potential harm. Furthermore, MR can be used to explore a wide range of exposures and outcomes across large populations, offering insights that can be generalized to broader populations.

Our study has several strengths. This study represents the first MR analysis to comprehensively investigate the causal relationships among SB, PA, and inflammation. Additionally, we examined several inflammatory markers, including the novel marker GlycA, which may better reflect chronic inflammation. Moreover, in our MR analysis, we used strong IVs, making weak IV bias unlikely. However, our study has some limitations. First, some IVs used in the MR analyses (such as causal measurements from television watching to CRP) displayed heterogeneity and may be associated with residual or unmeasured confounders. Second, this study focused on participants of European ancestry, highlighting the need for future research using GWAS data from diverse ethnic populations to enhance generalizability. Third, our findings indicate that different SB may affect inflammation differently; however, the underlying mechanisms may require further investigation. Fourth, we acknowledge additional potential confounders, such as body height, posture, and smoking. Furthermore, we recognize that television watching is often associated with unhealthy habits that may affect overall health. In our analysis, we selected BMI, waist circumference, hip circumference, waist-hip ratio, and body fat-related traits as confounders because of their significant impact on SB and PA. Although it is challenging to account for all possible confounders, the large sample size enhances the robustness and generalizability of our findings. Last, while our study highlights the positive causal effect of television watching as a type of SB on inflammation, it remains unclear whether varying types, intensities, and frequencies of sedentary interruptions have distinct effects on inflammatory markers, necessitating further research. GlycA emerges as a robust marker of chronic low-grade inflammation and may prove valuable in future studies.

## Conclusions

Using bidirectional two-sample MR analysis, we overcame the limitations of observational studies. Our findings demonstrated a potential causal relationship between television watching and chronic low-grade inflammation, with substantial evidence for GlycA, which may be a potential SB biomarker. Additionally, our study indicates that different types of SB may have varying and sometimes opposing effects on inflammation. Moreover, the correlation among SB, PA, and inflammatory markers may be partially or wholly driven by obesity-related traits. Furthermore, our findings underscore SB as an independent risk factor for inflammation compared to PA, offering insights into the distinct mechanisms through which SB and PA affect disease.

## Supporting information

S1 Fig Forest plots of television watching to GlycA, CRP, and CRP to “number of days/week of moderate PA 10+ minutes”. (A) Forest plot of television watching to GlycA. (B) Forest plot of television watching to CRP. (C) Forest plot of CRP to “number of days/week of moderate PA 10+ minutes.” GlycA: glycoprotein acetylation, CRP: C-responsive protein, PA: physical activity, SNP: single nucleotide polymorphism, MR: mendelian randomization.
(TIF)

S2 Fig Leave-one-out sensitivity analysis of television watching to GlycA, CRP, and CRP to “number of days/week of moderate PA 10+ minutes”. (A) Leave-one-out sensitivity analysis of television watching to GlycA. (B) Leave-one-out sensitivity analysis of television watching to CRP. (C) Leave-one-out sensitivity analysis of CRP to “number of days/week of moderate PA 10+ minutes.” GlycA: glycoprotein acetylation, CRP: C-responsive protein, PA: physical activity, SNP: single nucleotide polymorphism, MR: mendelian randomization.
(TIF)

S3 Fig Funnel plots of television watching to GlycA, CRP, and CRP to “number of days/week of moderate PA 10+ minutes”. (A) Funnel plot of television watching to GlycA. (B) Funnel plot of television watching to CRP. (C) Funnel plot of CRP to “number of days/week of moderate PA 10+ minutes.” GlycA: glycoprotein acetylation, CRP: C-responsive protein, PA: physical activity, SNP: single nucleotide polymorphism, MR: mendelian randomization.
(TIF)

S1 File S1-S10 Tables are included in file. (XLSX)
