# Supplementary figures and images for: Sedentary behavior from television watching elevates GlycA levels: A bidirectional Mendelian randomization study
Source: PLoS One. 2024 Aug 1;19(8):e0308301. doi: 10.1371/journal.pone.0308301 (PMC11293667; doi:10.1371/journal.pone.0308301)

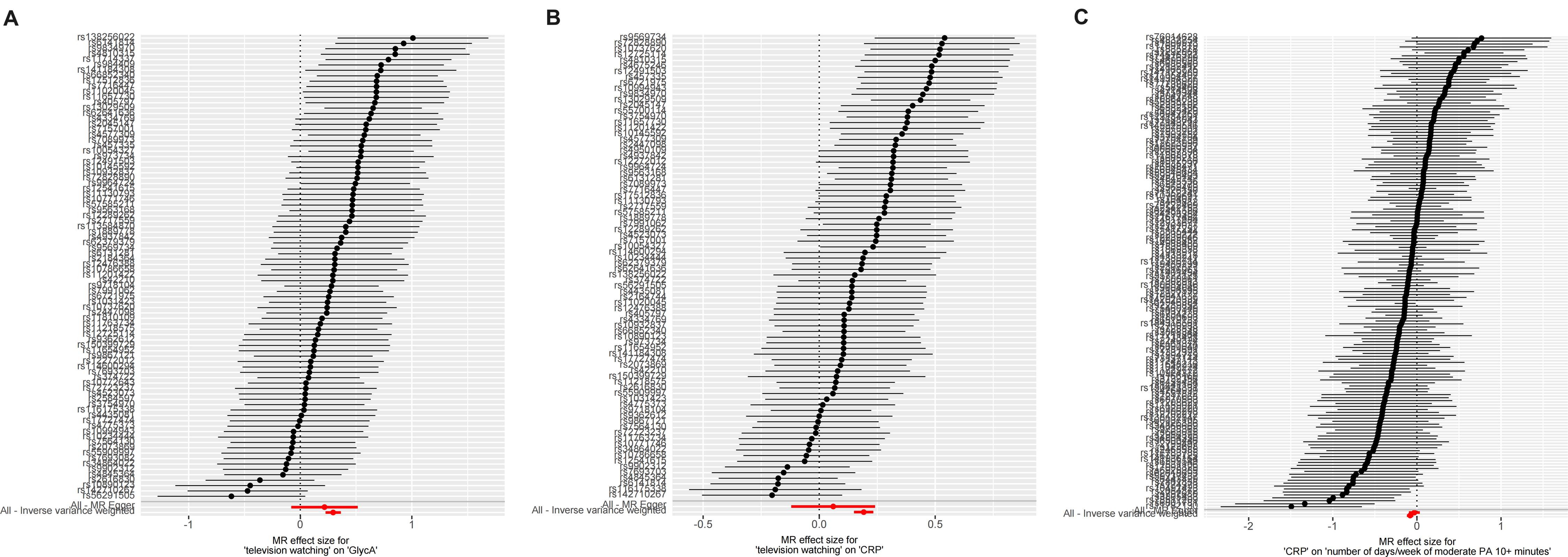

Supplement: S1 Fig — (A) Forest plot of television watching to GlycA. (B) Forest plot of television watching to CRP. (C) Forest plot of CRP to “number of days/week of moderate PA 10+ minutes.” GlycA: glycoprotein acetylation, CRP: C-responsive protein, PA: physical activity, SNP: single nucleotide polymorphism, MR: mendelian randomization. (TIF) [file pone.0308301.s001.tif]

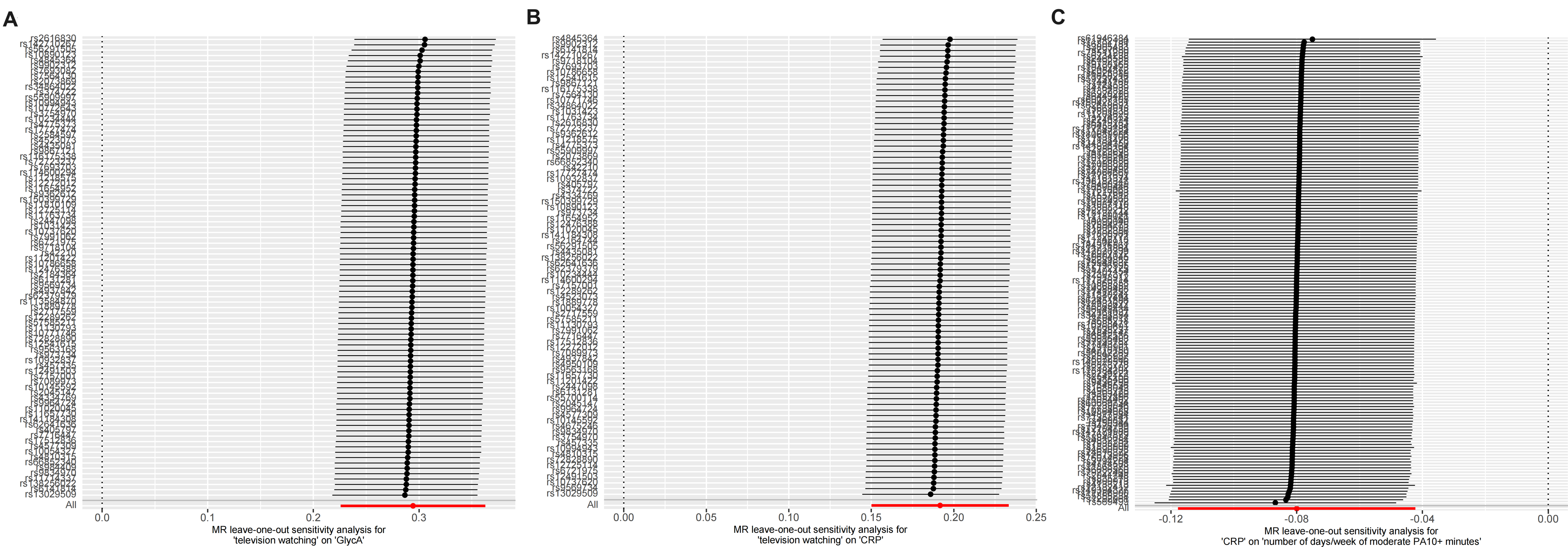

Supplement: S2 Fig — (A) Leave-one-out sensitivity analysis of television watching to GlycA. (B) Leave-one-out sensitivity analysis of television watching to CRP. (C) Leave-one-out sensitivity analysis of CRP to “number of days/week of moderate PA 10+ minutes.” GlycA: glycoprotein acetylation, CRP: C-responsive protein, PA: physical activity, SNP: single nucleotide polymorphism, MR: mendelian randomization. (TIF) [file pone.0308301.s002.tif]

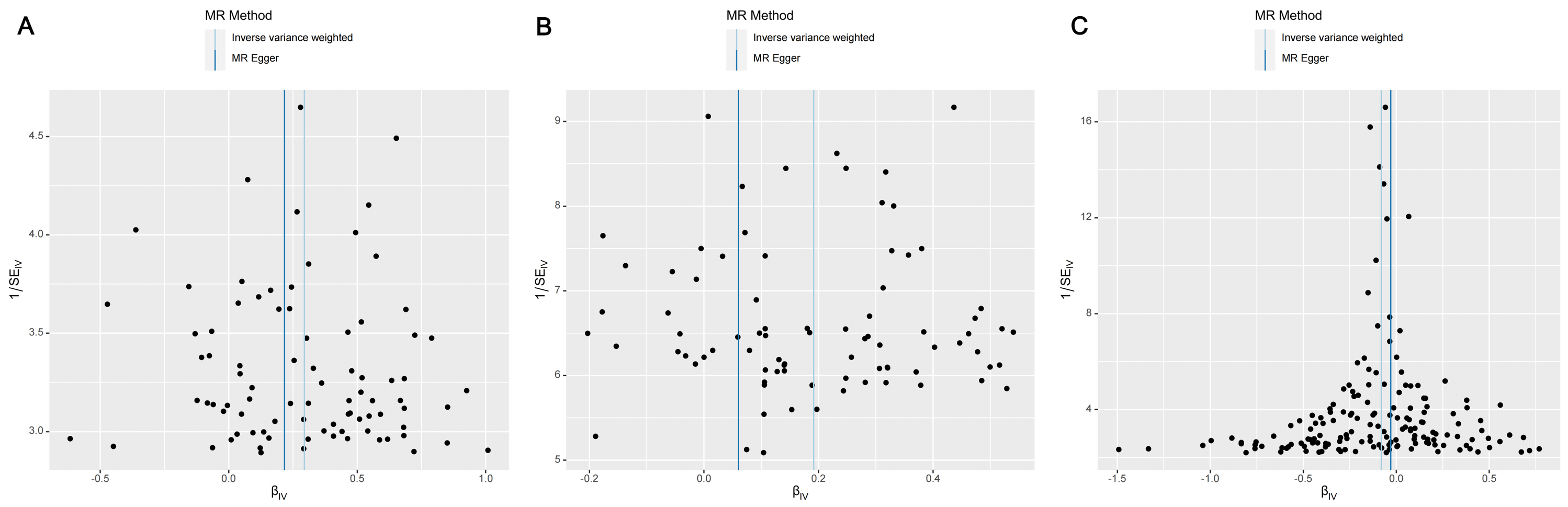

Supplement: S3 Fig — (A) Funnel plot of television watching to GlycA. (B) Funnel plot of television watching to CRP. (C) Funnel plot of CRP to “number of days/week of moderate PA 10+ minutes.” GlycA: glycoprotein acetylation, CRP: C-responsive protein, PA: physical activity, SNP: single nucleotide polymorphism, MR: mendelian randomization. (TIF) [file pone.0308301.s003.tif]
